# Supplementary material for: Observation of a three-dimensional quasi-long-range electronic supermodulation in YBa2Cu3O7−x/La0.7Ca0.3MnO3 heterostructures
Source: Nat Commun. 2016 Mar 1;7:10852. doi: 10.1038/ncomms10852 (PMC4773509; doi:10.1038/ncomms10852)
Supplement: Supplementary Information — Supplementary Figures 1-4 and Supplementary Note 1 [file ncomms10852-s1.pdf]

*Supplementary Information for*

# Observation of a Three-Dimensional Quasi-Long-Range Electronic Supermodulation in $YBa_2Cu_3O_{7-x}/La_{0.7}Ca_{0.3}MnO_3$ Heterostructures

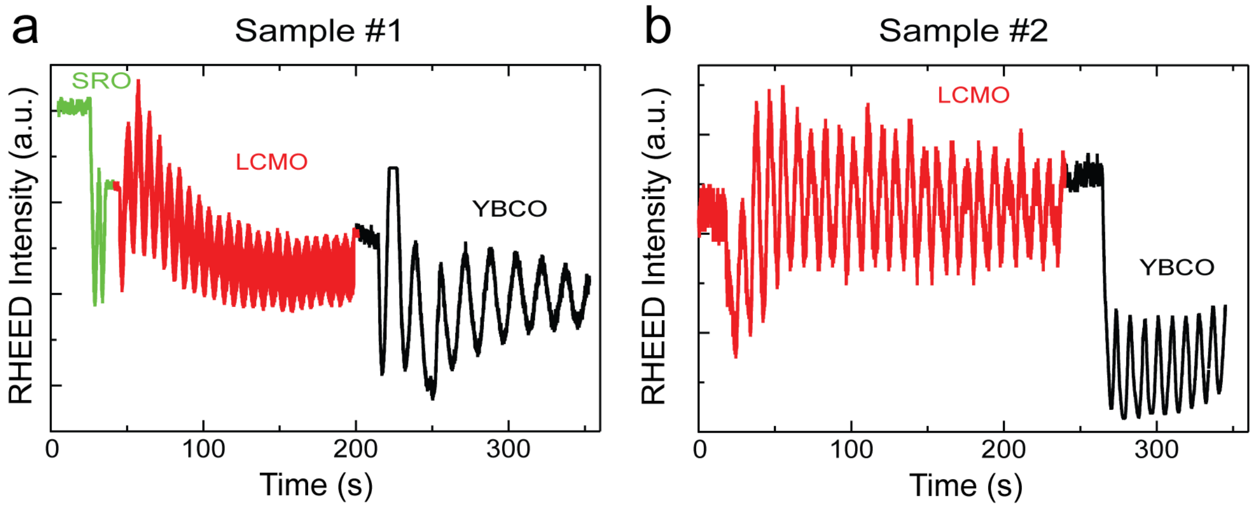


**Supplementary Figure 1: Layer-by-layer growth of the oxide layers.** Reflection high-energy electron diffraction (RHEED) oscillations recorded during the growth of the $La_{0.7}Ca_{0.3}O$-terminated (**a**) and $MnO_2$-terminated (**b**) samples.

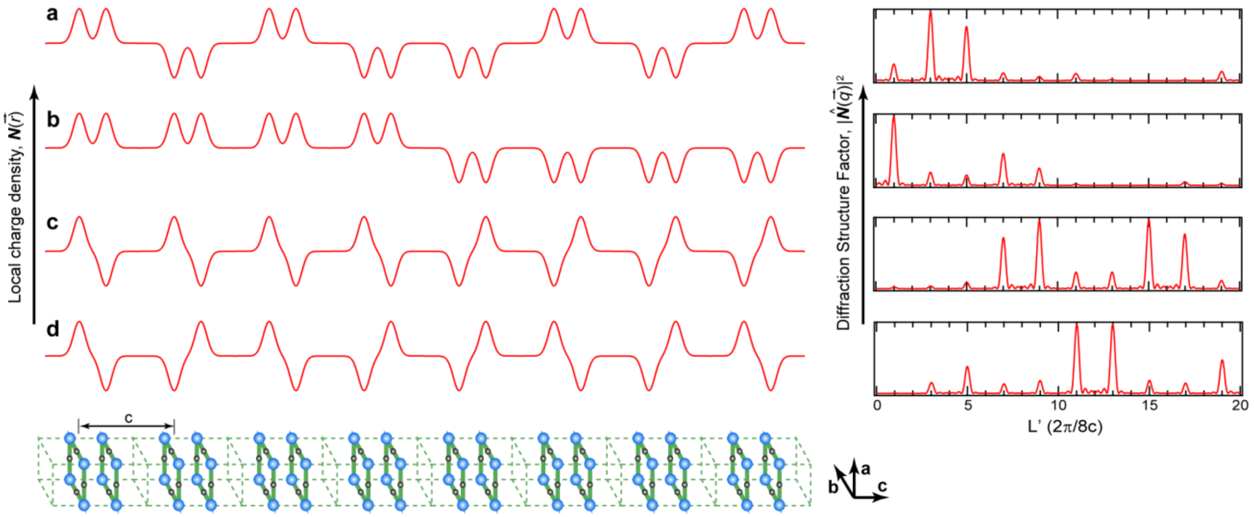


**Supplementary Figure 2: Calculation of diffraction structure factor for period-8 charge density modulations in different forms.** Four types of period-8 charge density modulations, $N(\vec{r})$ are considered (left column), with in-phase (**a** & **b**) or out-of-phase (**c** & **d**) modulation of the intra-bilayer charge density and in-phase (**b** & **c**) or out-of-phase (**a** & **d**) modulation of the inter-bilayer charge density. 25 u.c. are used to model each modulation. Diffraction structure factors (right column) are calculated as $|\widehat{N}(\vec{q})|^2$, where $\widehat{N}(\vec{q})$ is the Fourier transform of $N(\vec{r})$. Note the presence of a peak at L'=11 (in 2π/8c) and the absence at L'=10 and L'=12 in all cases. Results of **b** are reproduced in Main Fig. 2c.

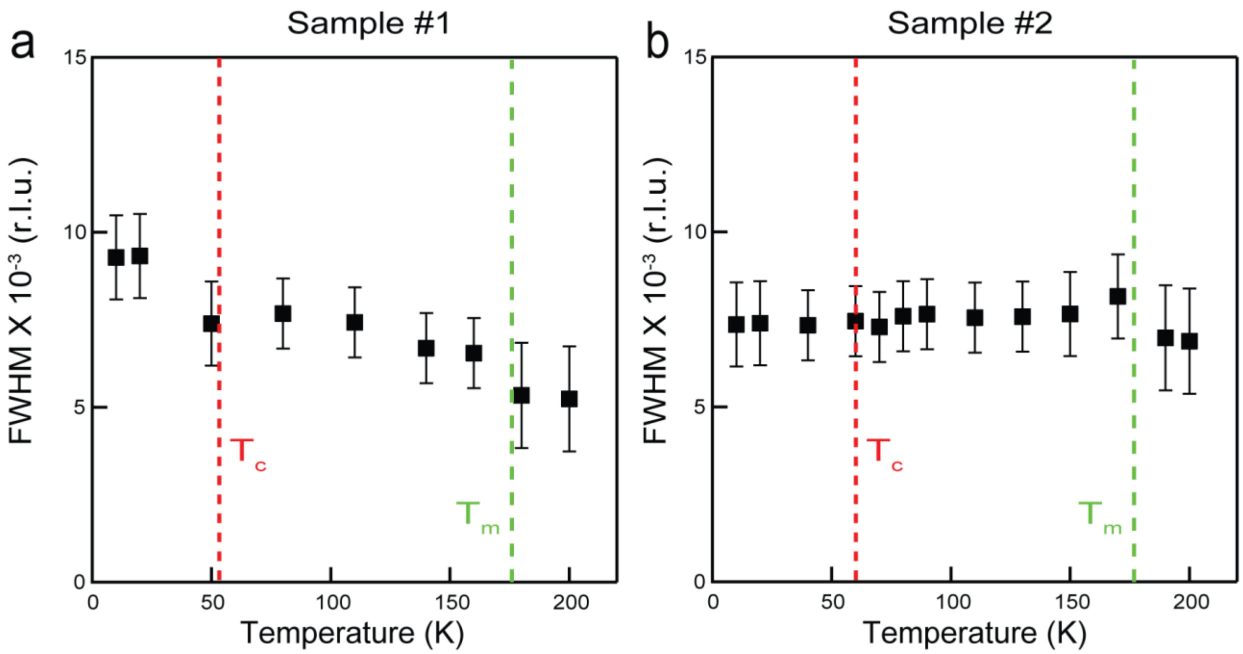


**Supplementary Figure 3: Temperature dependence of the full width half maximum (FWHM) of the diffraction peak measured on two samples.** The FWHM at each temperature is given by fitting the diffraction peak (as shown in Main Fig. 3) with a Lorentzian plus a constant background. The error bars reflect the uncertainty due to mean-square deviation in the peak fitting.

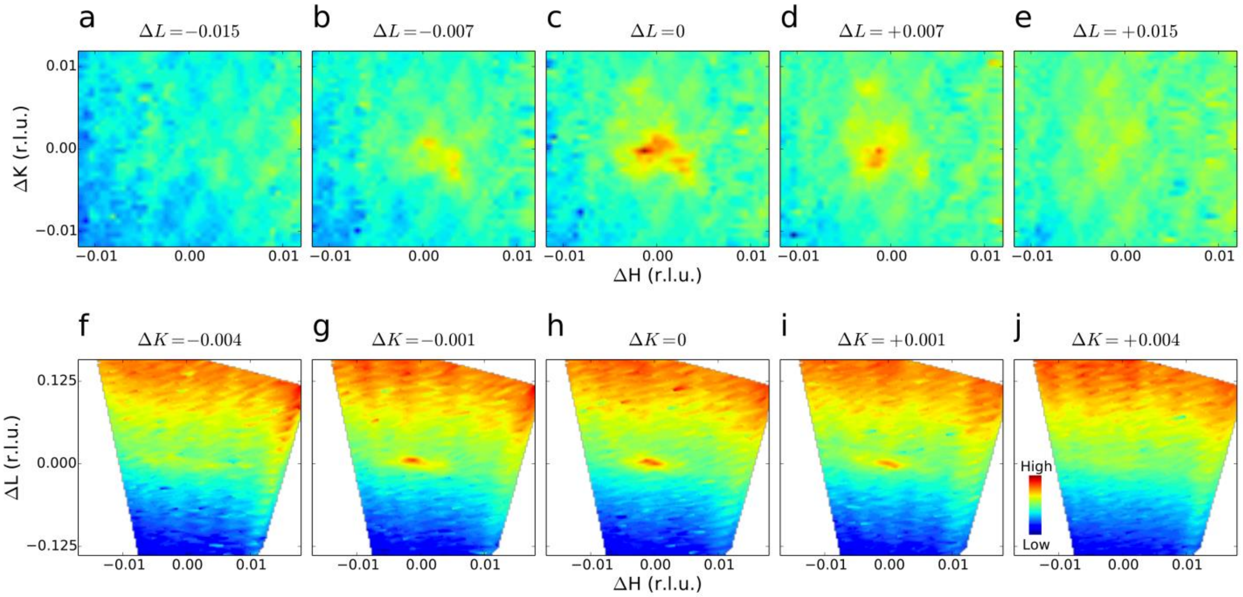


**Supplementary Figure 4: Reciprocal space maps of the supermodulation diffraction peak.** The three-dimensional nature of the supermodulation is verified by examining the reciprocal space volume around the corresponding diffraction peak. Coordinates are defined relative to the center of the diffraction peak, such that (*ΔH, ΔK, ΔL*) = (*H, K, L*) – (-0.245, 0, 1.38). *Top row*: Plan-view slices (*H-K* maps) at *ΔL* = **(a)** -0.015, **(b)** -0.007, **(c)** 0, **(d)** +0.007, and **(e)** +0.015. *Bottom row*: Cross-sectional slices (*H-L* maps) at *ΔK* = **(f)** -0.004, **(g)** -0.001, **(h)** 0, **(i)** +0.001, and **(j)** +0.004. The background level of intensity far from the peak center can be seen in **a** & **e** for the *H-K* maps, including a quasi-periodic pattern that is intrinsic to the CCD detector. Similarly, **f** & **j** display mainly the background level of intensity for the *H-L* maps; the strong linear background can also be seen in Main Fig. 2a. Data was measured on Sample #1 at 50 K.

**Supplementary Note 1:** In the main text we presented a simplified data processing method for extracting REXS intensity from the CCD images, essentially treating the CCD as a selected area point detector. Although a point detector can miss significant features—including the 3D shape of a diffraction peak—when insufficient reciprocal volume has been sampled, we have verified the validity of the simplified method for the current study. Accordingly we have constructed reciprocal space maps (RSMs) from the full CCD pixel array that provide a view of the 3D reciprocal volume measured by each scan.

Supplementary Fig. 4 shows several representative slices of reciprocal space (*H-K* maps at various *L*, as well as *H-L* maps at various *K*) that cover the extent of the diffraction peak. A strong diffraction peak appears in the *H-K* map at $L$ = 1.38 (r.l.u.). This peak becomes weaker and disappears as *L (*or *H or K)* moves farther away from its center value ($\Delta L = \Delta H = \Delta K = 0$). Notably the intensity of the diffraction peak diminishes appreciably in every direction away from the peak center, and no rod-like feature is observed along any direction. Therefore we can conclude that the supermodulation we observe by REXS truly exhibits three-dimensional order. Additionally we note that the line scans in Main Figs. 1 & 2 that were compiled by the simplified method described earlier are indeed valid and equivalent to results obtained from full RSMs because the only notable intensity peak found in our scans is localized around the supermodulation ordering vector.
